# Liver Transplantation for Refractory Congenital Cytomegaloviral Hepatitis

**DOI:** 10.1097/PG9.0000000000000275

**Published:** 2022-12-02

**Authors:** David D. Aufhauser, Paige Condit, Kathryn M. Schmit, James H. Conway, Shelly Cook, Anthony M. D’Alessandro, Katryn N. Furuya

**Affiliations:** From the * Department of Surgery, University of Wisconsin-Madison School of Medicine and Public Health, Madison, WI; † Department of Pediatrics, University of Wisconsin- Madison School of Medicine and Public Health, Madison, WI.

**Keywords:** cytomegalovirus hepatitis, live donor liver transplantation, liver transplantation

## Abstract

Congenital cytomegalovirus (cCMV) is the most common congenital infection. Here, we report on a case of severe, refractory cCMV hepatitis resulting in end-stage liver disease. A male infant born at 37 weeks gestational age presented with petechiae, splenomegaly, and jaundice associated with a direct hyperbilirubinemia, elevated transaminases, and thrombocytopenia. Urine screen was positive for CMV, and he was treated with valganciclovir. He progressed to decompensated cirrhosis with ascites, hypoglycemia, and coagulopathy and was listed for liver transplant at 4 months of age. At 5 months of age, he developed massive hematemesis with hemorrhagic shock and underwent emergent portocaval shunt followed by living donor liver transplant with a left lateral segment graft. Postoperatively, he received CMV immune globulin and intravenous ganciclovir and cleared his viremia by 2 months post-transplant. This case illustrates the diagnostic and management challenges of severe cCMV hepatitis and reports a successful liver transplantation despite active CMV viremia.

## INTRODUCTION

Congenital cytomegalovirus (cCMV) is the most common congenital infection effecting 0.5%–1% of live births in the United States with 11% of these being symptomatic infections (1). CMV has tropism to the reticuloendothelial system, and 7%–75% of symptomatic neonates have hepatic involvement (2,3). Hepatic manifestations of cCMV in most infants typically resolve spontaneously or with antiviral treatment (2,4). There are infrequent reports of cCMV leading to liver failure before the introduction of ganciclovir in 1988 (5,6), but a recent report suggests that cCMV leading to liver failure despite antiviral therapy may be more common than is recognized (7). We report a patient with liver failure due to cCMV who was successfully treated with a living donor liver transplant.

## CASE REPORT

**FIGURE 1.**
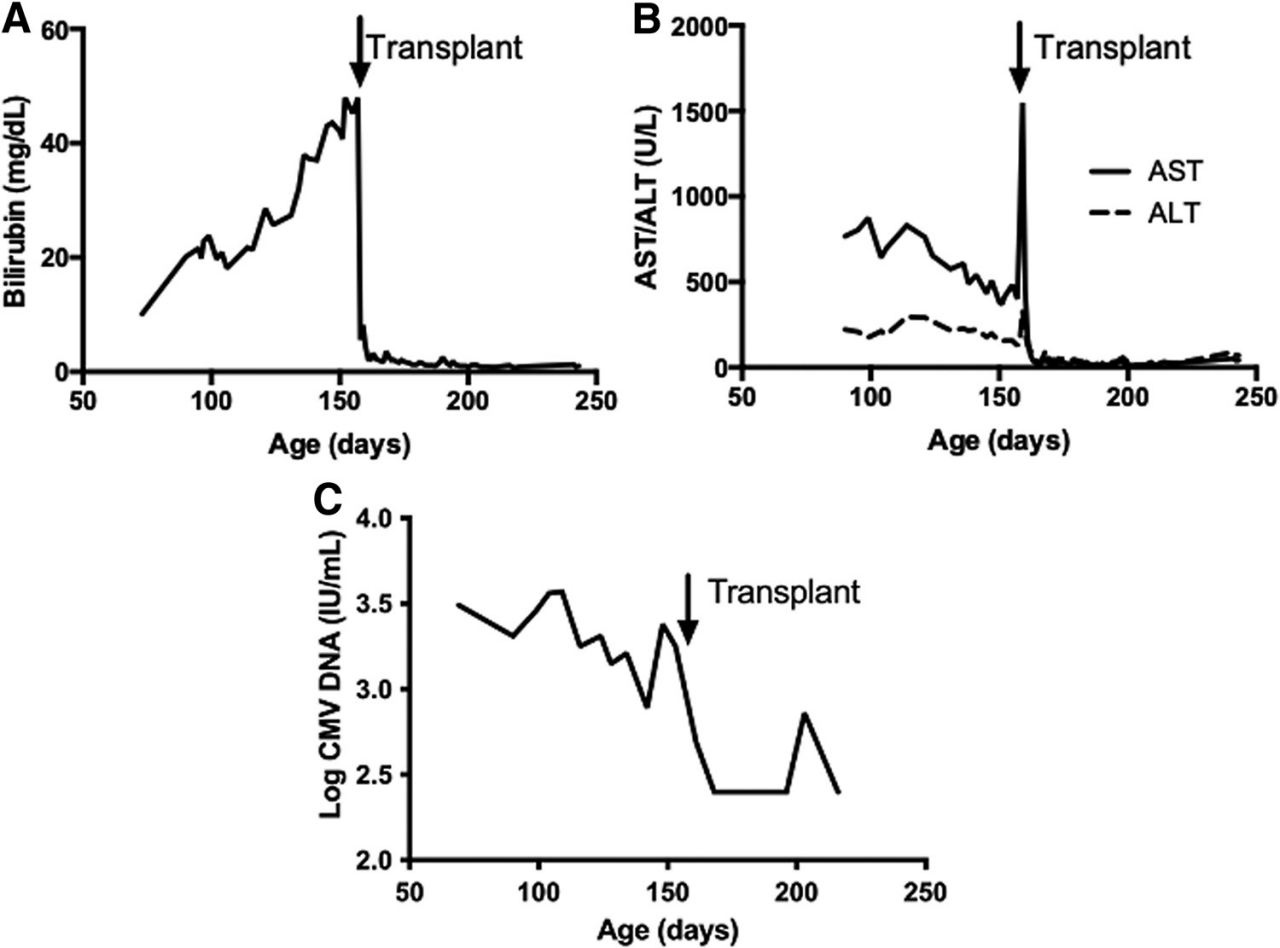
Laboratory parameters. Trends in patient’s serum total bilirubin (A), serum AST and ALT (B), and serum CMV quantification (C) from presentation through transplant. ALT = alanine transaminase; AST = aspartate transaminase; CMV = cytomegalovirus.

A 2.5 kg male born at 37 weeks gestational age to a gravida 2, para 2 Caucasian mother presented with petechiae, splenomegaly, and jaundice at an outside institution. The patient’s initial laboratory values included total bilirubin 8.2 mg/dL (reference 0.0–1.3 mg/dL), gamma-glutamyltransferase 154 U/L (reference 0–40 U/L), and platelet count 21 k/γL (reference 140–385 k/γL) (Fig. 1A, B). Birth weight was at the 3rd percentile and head circumference at the 13th percentile. A urine CMV polymerase chain reaction was positive on the first day of life, and oral valganciclovir was initiated. Ophthalmologic examination, audiology evaluation, and head imaging did not identify any additional CMV-associated manifestations.

**FIGURE 2.**
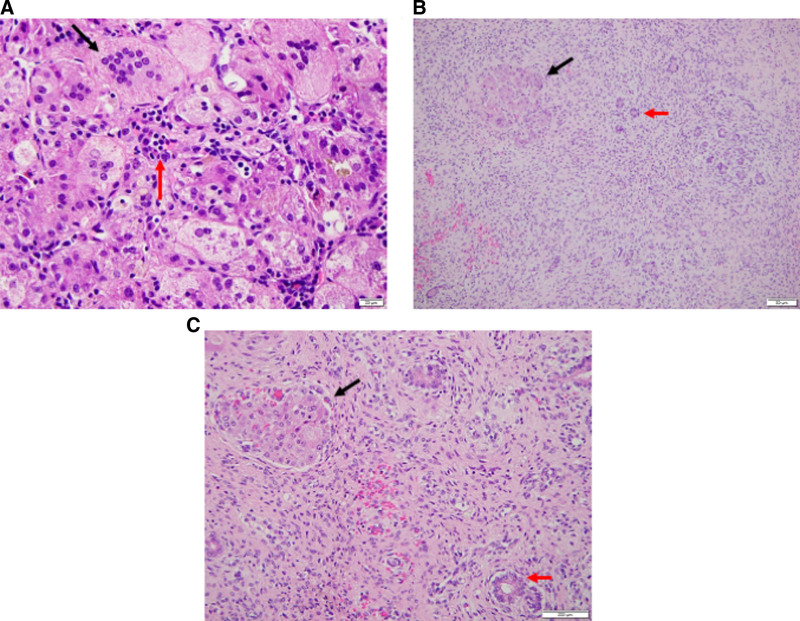
Histology. A) Native liver biopsy demonstrated giant cell hepatitis with prominent large multinucleated hepatocytes (black arrow) and scattered extramedullary hematopoiesis (red arrow). B and C) Explant pathology revealing pan-lobular necrosis with severe parenchymal collapse (black arrow) and fibrosis with small nests of residual hepatocytes (red arrow).

Despite antiviral treatment, the patient had persistent CMV viremia (Fig. 1C), hepatosplenomegaly, hepatitis, and hyperbilirubinemia. A 66-gene genetic cholestasis panel (EGL Genetics, Tucker, GA) was negative for abnormalities. Serologies against hepatitis B and C, HIV, and Epstein-Barr virus were negative. At 2 months of age, hepatobiliary scintigraphy showed no excretion of radiotracer into the extrahepatic bile ducts or small bowel, raising suspicion of biliary atresia. Percutaneous cholangiogram, however, showed normal extrahepatic bile ducts with free flow of bile into the duodenum. Percutaneous biopsies demonstrated giant cell hepatitis with prominent large multinucleated hepatocytes and scattered extramedullary hematopoiesis but no CMV on immunohistochemistry (Fig. 2A). Next-generation sequencing on a plasma CMV sample did not demonstrate UL97 or UL54 gene mutations, which are seen in antiviral resistant disease.

The patient’s liver failure continued to progress. At 3 months of age, he developed decompensated cirrhosis with ascites, hypoglycemia requiring continuous nasogastric feeding, and coagulopathy. His CMV viral load remained elevated, and he was transitioned to intravenous ganciclovir and given a dose of CMV immune globulin. He was listed for liver transplant, initially with a Pediatric End-Stage Liver Disease (PELD) score of 28 that eventually progressed to a PELD score of 40. Evaluation of potential living donors was initiated, and an unrelated compatible donor was identified.

At 5 months of age and on the morning of a planned live donor liver transplant, the patient developed massive hematemesis with hemorrhagic shock. He was emergently taken to the operating room (OR) for a portocaval shunt. He was resuscitated postoperatively. A computerized tomography scan of his head was obtained to aid in neurologic prognostication. It revealed a small epidural hematoma that was stable on follow-up imaging at 1 hour and 12 hours intervals. The patient’s prognosis and the possibility of a poor outcome following transplant were discussed with the patient’s parents, the living donor, the transplant team, and neurosurgical consultants, and the donor opted to proceed after being fully informed of the risks. The patient returned to the OR the following day for living donor transplant with a left lateral section graft. His explant pathology revealed pan-lobular necrosis with severe parenchymal collapse, fibrosis, and small nests of residual hepatocytes (Fig. 2B, C).

The patient received corticosteroids and tacrolimus with an initial goal trough level of 5–7 for immunosuppression. He received 2 doses of CMV immune globulin on postoperative days 1 and 14 and was continued on intravenous ganciclovir. By post-transplant day 9, his CMV DNA had fallen to <250 copies/mL. He subsequently had a single detectable CMV viral load that resolved without change to therapy. He was transitioned to oral valganciclovir twice daily at 2 months post-transplant. Valganciclovir was discontinued at 12 months post-transplant, and the patient was transitioned to CMV periodic monitoring. He is now 18 months post-transplant doing well with an undetectable CMV DNA. He has had no episodes of rejection.

## DISCUSSION

This case of an infant with cCMV causing end-stage liver disease treated with a successful liver transplantation during active CMV viremia illustrates many of the diagnostic and management challenges of severe cCMV. Hepatic manifestations of cCMV are relatively common, and recent reports suggest that liver failure from cCMV may be unrecognized (2–7). It is unclear why this infant had developed such an aggressive and refractory case of cCMV. Next-generation sequencing was performed relatively early in the patient’s disease course and did not identify any common mutations associated with antiviral resistance. An immunodeficiency evaluation with lymphocyte subset analysis with flow cytometry did not identify a T-cell deficiency or natural killer cell deficiency that could explain his persistent infection, and the patient did not exhibit evidence of extrahepatic disease. We hypothesize that his refractory viremia was due to a high burden of disease in the liver that acted as a viral reservoir despite antiviral treatment. The hepatectomy at time of transplant essentially debrided this reservoir and facilitated post-transplant CMV viral clearance despite immunosuppression. Although his explant liver pathology did not identify viral inclusions and his CMV immunohistochemistry was negative, these findings are in line with other reports of difficulty in identifying virus on histology in cCMV hepatitis and may reflect the limit of histopathology in severe liver necrosis (7).

Our initial strategy, in this case, aimed to clear the patient’s viremia in the hopes of averting the need for transplant and decreasing any potential risk of CMV recurrence after transplant. This approach was revised as the patient’s liver function rapidly deteriorated requiring an urgent need for transplantation. The case illustrates that liver transplant for severe cCMV liver failure can be successfully performed in the presence of CMV viremia and that transplant may even facilitate viral clearance despite post-transplant immunosuppressive regimens.
